# Patient-reported measures of well-being in older multiple myeloma patients: use of secondary data source

**DOI:** 10.1007/s40520-019-01465-3

**Published:** 2020-01-22

**Authors:** Irena Cenzer, Karin Berger, Annette M. Rodriguez, Helmut Ostermann, Kenneth E. Covinsky

**Affiliations:** 1 Division of Geriatrics, University of California, San Francisco, 4150 Clement St., San Francisco, CA 94121 USA; 2 Department of Medicine III, University Hospital, LMU Munich, Munich, Germany; 3 Veterans Affairs Medical Center, San Francisco, CA USA; 4 Medizinische Klinik und Poliklinik III, Health Care Research, Outcomes Research and Health Economics, Marchioninistraße 15, 81377 Munich, Germany

**Keywords:** Patient-reported measures, Multiple myeloma, Geriatric assessment, Secondary data use, Rare diseases

## Abstract

**Background:**

Changes in well-being of patients with multiple myeloma (MM) before and after diagnosis have not been quantified.

**Aims:**

Explore the use of secondary data to examine the changes in the well-being of older patients with MM.

**Methods:**

We used the Health and Retirement Study (HRS), linked to Medicare claims to identify older MM patients. We compared patient-reported measures (PRM), including physical impairment, sensory impairment, and patient experience (significant pain, self-rated health, depression) in the interviews before and after MM diagnosis using McNemar’s test. We propensity-matched each MM patient to five HRS participants without MM diagnosis based on baseline characteristics. We compared the change in PRM between the MM patients and their matches.

**Results:**

We identified 92 HRS patients with MM diagnosis (mean age = 74.6, SD = 8.4). Among the surviving patients, there was a decline in well-being across most measures, including ADL difficulty (23% to 40%, *p* value = 0.016), poor or fair self-rated health (38% to 61%, *p* value = 0.004), and depression (15% to 30%, *p* value = 0.021). Surviving patients reported worse health than participants without MM across most measures, including ADL difficulty (40% vs. 27%, *p* value = 0.04), significant pain (38% vs. 22%, *p* value = 0.01), and depression (29% vs. 11%, *p* value = 0.003).

**Discussion:**

Secondary data were used to identify patients with MM diagnosis, and examine changes across multiple measures of well-being. MM diagnosis negatively affects several aspects of patients’ well-being, and these declines are larger than those experienced by similar participants without MM.

**Conclusion:**

The results of this study are valuable addition to understanding the experience of patients with MM, despite several data limitations.

## Introduction

Multiple myeloma is a rare disease, affecting approximately 7 out of 100,000 persons in the United States each year. Multiple myeloma disproportionately affects older patients, with a median age at diagnosis being over 70 years old [1]. While new treatments are increasing the survival time [2], we know less about the quality of life of older persons who are more susceptible to treatment related adverse events. Older patients are concerned not only with survival time, but also with symptom management, maintaining independence, and how their well-being will be affected by the disease and treatments [3]. Previous studies have shown that patients with multiple myeloma experience higher symptom burden and lower quality of life compared to the general population [4, 5], but these studies did not focused on older patients specifically. Overall, there is a consensus that older patients with multiple myeloma could benefit from a geriatric assessment before and during treatment [3, 6, 7]. However, to our knowledge, there have been no studies that formally quantify patient-reported measures in older multiple myeloma patients before and after the diagnosis, or studies comparing the changes in well-being to older adults experiencing the normal ageing process.

As with any rare disease, it can be difficult to recruit large numbers of multiple myeloma patients for research studies due to the low prevalence of the disease, which makes recruiting large numbers of patients resource intensive. One potential source of data for studies of rare diseases is secondary data, including large observational studies and administrative data. In this study we explore studying the well-being of older multiple myeloma patients using Medicare claims data, and Health and Retirement Study (HRS), an ongoing longitudinal study started in 1992. The use of this longitudinal cohort has several advantages. First, due to length of the study period, enough cases accrue over time, creating a feasible study sample. Second, HRS is a detailed study with data on a variety of health status measures that are of interest for older patients. Third, since HRS is a longitudinal study, each surviving participant is interviewed every 2 years, meaning that the measures of interest are observed before and after the diagnosis. Fourth, we can compare changes in the health status measures in HRS participants without multiple myeloma, for whom we have the same health status measures over the same period of time.

The use of HRS data that were not collected for the purpose of learning about well-being of multiple myeloma patients imposes limits on questions that can be answered. For example, since we do not have the information on what treatment the patients receive, we can not determine if well-being changes are due to the disease or its treatment. In addition, because the HRS interviews are biennial, the time between multiple myeloma diagnosis and report of health status measures varies between 0 days and 2 years. However, HRS data also make it possible to address issues that would be difficult in a primary data collection study. It makes it possible to collect data on prediagnosis health status rather than retrospective reports. In addition, we can compare changes in health to population-representative subjects without multiple myeloma.

We conducted this study examining changes before and after multiple myeloma diagnosis with three specific goals: (1) assessing feasibility of using claims data to identify and study patients with multiple myeloma; (2) assessing the change in patient-reported measures before and after diagnosis; (3) comparing the change in patient-reported measures to a comparable sample of individuals without multiple myeloma.

## Methods

### Participants

The Health and Retirement Study (HRS) was designed to examine changes in health and wealth as people age [8]. It is an ongoing nationally representative longitudinal study of participants age 50 and older. The study started in 1992 and follow up surveys are administered every 2 years. If a participant is not able to complete an interview, the interview is conducted with a proxy respondent.

We examined participants who had a diagnosis of multiple myeloma while enrolled in the HRS, ascertained by linking the HRS survey data to Medicare claims. A participant was identified as having a diagnosis of multiple myeloma if he or she had two or more Medicare claims with ICD9 code 203.0. While we required two claims to confirm the diagnosis of multiple myeloma, the date of diagnosis was set to the date of first claim.

Out of 28,927 HRS participants age 65 or older at any point between 1992 and 2015, 26,044 (90.0%) agreed to have their HRS surveys linked to the Medicare claims. We identified 126 participants who had two or more claims with multiple myeloma. Since we wanted to ensure that the first claim observed in our files was indeed the first diagnosis of multiple myeloma, we excluded 21 (16.7%) participants if they were not enrolled continuously in fee-for-service Medicare for 6 months prior to the first observed claim with multiple myeloma diagnosis. Of the remaining 105 multiple myeloma patients, 13 (12.4%) did not complete a HRS interview within 3 years before a multiple myeloma diagnosis and were, therefore, excluded. This resulted in a sample of 92 multiple myeloma patients included in the study.

### Measures

All baseline characteristics of study participants were taken from the last HRS interview before a diagnosis of multiple myeloma. Those included participant characteristics (age, gender, race, marital status, education, wealth, comorbidities, and health behaviors), and patient-reported measures of interest.

We considered nine patient-reported measures that cover a range of well-being for multiple myeloma patients, and are part of geriatric assessment of older patients [9, 10]. The included measures cover the domains that matter most to patients: functioning, sensory impairment, and general well-being. While the nine measures do not constitute together a validated questionnaire, each one of the measures has been validated or used in prior research of patients’ well-being. We include below the exact text of the survey question for each measure, and the justification for the new coding, when the questions were not used in the original form. Additionally, HRS website includes detailed documentation guides for all the measures used in this study [8]. Four measures describe limitations in physical functioning: difficulty in any Activities of Daily Living (ADL), difficulty in any Instrumental Activities of Daily Living (IADL), difficulty walking several blocks, and difficulty climbing one flight of stairs. ADL and IADL measures are standardized measures of assessing function in older adults in both clinical and research setting [11, 12]. Difficulty in any functional measure was assessed in the HRS by asking, “Because of a health or memory problem do you have any difficulty with [activity]?”. There were six ADLs (bathing, eating, walking across the room, transferring to and from bed, using toilet, and dressing), and five IADLs (using a phone, preparing hot meals, grocery shopping, managing financing, managing medications) included in the assessment. Next, two sensory impairment measures were included: vision and hearing impairment. Each of the two sensory impairments were assessed by asking, “Is your [hearing/eyesight] excellent, very good, good, fair, or poor using a [hearing aid/glasses or corrective lenses] as usual?”. Finally, three additional measures of general well-being were considered: experience of significant pain, self-rated health, and self-reported symptoms of depression. The presence of significant pain was determined using two questions. First, subjects were asked, “Are you often troubled with pain?”. Subjects who responded “Yes” were then asked, “How bad is the pain most of the time: mild, moderate or severe?” Subjects who responded “moderate” or “severe” were classified as experiencing significant pain. This classification of significant pain has been applied in previous studies [13, 14], because it reflects the American Geriatrics Society Guidelines for the Pharmacologic Management of Persistent Pain in Older Adults and the National Comprehensive Cancer Network Clinical Practice Guidelines in Oncology, which both recommend that moderate or severe pain should prompt a clinical response [15, 16]. The SUPPORT study (Study to Understand Prognoses and Preferences for Outcomes and Risks of Treatments) also used this classification to categorize reports of pain at the end of life [17]. Symptoms of depression were measured using the eight-item Center for Epidemiologic Studies Depression Scale [18, 19]. Participants were classified as having symptoms consistent with the diagnosis of depression if they reported more than three symptoms.

The same nine patient-reported measures were also assessed at the first HRS interview after a diagnosis of multiple myeloma.

### Statistical analysis

We started with describing the multiple myeloma patients in terms of demographics, socioeconomic status and health status. Next, we assessed the frequency of each patient-reported measure before a multiple myeloma diagnosis. Depression was not assessed for participants with proxy respondents; therefore, 10 patients were not included in the analysis of frequency and changes in depression. Questions about pain levels was not asked in early HRS interviews (1992–1993), so 5 patients were not included in the analyses invlolving significant pain. Analyses were conducted independently for each variable to prevent missing values from one variable affecting the sample size in the analysis of other variables.

Next, we compared the frequency of adverse health on the patient-reported measures before and after a multiple myeloma diagnosis. Only patients that completed the interview after their multiple myeloma diagnosis were included in this analysis, as the goal of this study was to describe well-being and changes in well-being of the patients who survive the initial period after multiple myeloma diagnosis. Patients who died within 2 years of multiple myeloma diagnosis, but completed an HRS interview between their diagnosis and death, were included in the study. The comparison of patients before and after multiple myeloma diagnosis was done using McNemar’s test.

Finally, we compared the change in frequency of adverse health on the patient-reported measures between HRS patients with a diagnosis of multiple myeloma to those without the diagnosis over the same time period, i.e., selecting subjects interviewed around the same time. To perform this comparison, we matched each patient with multiple myeloma to five individuals without multiple myeloma. The matching was performed using a mixture of propensity score matching and exact matching. Patients were matched on propensity score for being diagnosed with multiple myeloma, calculated using age, gender, race, marital status, education, wealth, number of comorbidities, and smoking status. Additionally, patients were matched on exact year of interview before and after a diagnosis of multiple myeloma, and on exact status of patient-reported measure for the multiple myeloma patient before the diagnosis. The matching was done separately for each patient-reported measure. This analysis was again limited only those patients who completed the HRS interview after a diagnosis of multiple myeloma, as our goal was to describe the experience among surviving patients.

Statistical analyses were performed using SAS 9.4 [20] and R 3.4 [21, 22] software. This study has been approved by the Center for Disease Control’s Institutional Review Board (CDC).

## Results

Table 1 describes the prediagnosis baseline characteristics of the multiple myeloma patients included in this study. The mean age of 92 patients was 74.6 (SD = 8.4), and 47% were women. Forty-three percent of patients had 3 or more chronic conditions before diagnosis. The most common ADL difficulties were getting dressed (13%) and bathing (13%). The most common IADL difficulties shopping for groceries (17%) and preparing hot meals (17%). Eighteen patients (20%) died within 1 year, and 38 (41%) died within 2 years of multiple myeloma diagnosis. This mortality rate is comparable to mortality rates reported in other long-term studies of older adults [2, 23]. Of the 92 patients included in the study, 66 (72%) completed HRS interview after multiple myeloma diagnosis. Most subjects not completing an interview died before there scheduled interview. The median time between interview before multiple myeloma diagnosis and the diagnosis was 13.0 months (IQR 6.3–19.1), and the median time between the diagnosis and the first interview after the diagnosis was 12.6 months (6.0–18.0) (Table 1).

**Table 1:** Baseline characteristics of multiple myeloma patients before diagnosis

Characteristics	*N* (%)
Age	74.6 ± 8.4
Women	43 (47%)
White race	70 (76%)
Married	54 (59%)
Education less than High School	25 (27%)
Wealth (median, IQR)	151 K (45–405 K)
BMI	
≤ 25	31 (34%)
> 25	59 (66%)
Number of comorbidities ≥ 3	40 (43%)
Previous diagnosis of cancer	27 (30%)
Current smoker	6 (7%)
Drinker	41 (45%)
ADL difficulty	
Bathing	12 (13%)
Eating	5 (5%)
Walking across the room	9 (10%)
Transferring to and from bed	8 (9%)
Dressing	12 (13%)
Using toilet	6 (7%)
IADL difficulty	
Using the telephone	6 (7%)
Managing finances	13 (14%)
Managing medications	6 (7%)
Shopping for groceries	15 (17%)
Preparing meals	15 (17%)
Proxy interview	10 (11%)
Months between interview before diagnosis and diagnosis (median, IQR)	13.0 (6.3–19.1)
Months between diagnosis and interview after diagnosis (median, IQR)	12.6 (6.0–18.0)
Died within 1 year of diagnosis	18 (20%)
Died within 2 years of diagnosis	38 (41%)

*BMI* body mass index, *ADL* activities of daily living, *IADL* instrumental activities of daily living

About a quarter of all multiple myeloma patients reported difficulty with at least one ADL (*n* = 22, 24%) before the diagnosis (Table 2). Twenty-nine patients (32%) reported difficulty with at least one IADL, 34 patients (37%) reported difficulty with walking several blocks, and 25 patients (27%) reported difficulty with climbing one flight of stairs. Vision impairment was reported by 29 patients (32%), and hearing impairment was reported by 20 patients (22%) before multiple myeloma diagnosis. About a quarter of the patients experienced significant pain (*n* = 20, 23%), and about a fifth reported symptoms of depression (*n* = 15, 19%). The most common impairment before multiple myeloma diagnosis was poor or fair self-reported health (*n* = 37, 40%).

**Table 2:** Frequency of impairment on patient reported measures in the interview before multiple myeloma diagnosis

Characteristic	*N* (%)^a^
Physical impairment	
Difficulty with any ADL	22 (24%)
Difficulty with any IADL	29 (32%)
Difficulty with walking several blocks	34 (37%)
Difficulty with climbing one flights of stairs	25 (27%)
Sensory impairment	
Vision Impairment	29 (32%)
Hearing Impairment	20 (22%)
Patient experience	
Significant pain	20 (23%)
Poor self-rated health	37 (40%)
Depression	15 (19%)

^a^Number of missing values for each variable is as follows: Difficulty was any ADL (1). Difficulty with any IADL (2), Difficulty with walking several blocks (1), Difficulty with climbing one flight of stairs (1), Significant pain (6), Depression (11)

Impairments in patient-reported measures in surviving multiple myeloma patients increased significantly after the diagnosis (Table 3). Patients reported higher rates of difficulty in three of the four physical measures, including increases in ADL difficulty (23% to 40%, *p* = 0.02), difficulty walking several blocks (30% to 60%, *p* < 0.001), and difficulty climbing one flight of stairs (25% to 47%, *p* = 0.003). There was no statistically significant change in IADL difficulty (30% to 41%, *p* = 0.13) The rate of depression symptoms doubled after the diagnosis (15% to 30%, *p* = 0.02), and more patients reported poor or fair self-rated health (38% to 61%, *p* = 0.004). There was no significant increase in patients experiencing significant pain (27% to 38%, *p* = 0.09). There was no change at all in the rates of vision impairment (30% to 30%, *p* = 1), but patients reported significant increase in hearing impairment (20% to 33%, *p* = 0.04).

**Table 3:** Frequency of impairment on patient-reported measures in the interviews before and after multiple myeloma (MM) diagnosis, in patients who survived until HRS interview after diagnosis

Characteristic	*N* in comparison	Before MM diagnosis	After MM diagnosis	*p* value
Physical impairment				
Difficulty with any ADL	65	15 (23%)	26 (40%)	0.016
Difficulty with any IADL	64	19 (30%)	26 (41%)	0.127
Difficulty with walking several blocks	63	19 (30%)	38 (60%)	<0.001
Difficulty with climbing one flights of stairs	59	15 (25%)	28 (47%)	0.003
Sensory impairment				
Vision impairment	66	20 (30%)	20 (30%)	1.000
Hearing impairment	66	13 (20%)	22 (33%)	0.039
Patient experience				
Significant pain	64	17 (27%)	24 (38%)	0.090
Poor self-rated health	64	24 (38%)	39 (61%)	0.004
Depression	53	8 (15%)	16 (30%)	0.021

Matched HRS participants without a multiple myeloma diagnosis reported lower rates of some patient-reported measures in the interview after the multiple myeloma diagnosis of the patients (Table 4), even though they were matched to have the same level of impairment before the diagnosis. Participants without multiple myeloma reported lower rates in ADL difficulty (40% vs. 27%, *p* = 0.04), difficulty walking several blocks (60% vs. 37%, *p* < 0.001), and difficulty climbing one flight of stairs (47% vs. 29%, *p* = 0.005). There were no statistically significant differences in IADL difficulty (41% vs. 32%, *p* = 0.21), vision impairment (30% vs. 26%, *p* = 0.52), or hearing impairment (33% vs. 29%, *p* = 0.44). Multiple myeloma patients reported higher rates of poor or fair self-rated health (61% vs. 39%, *p* = 0.001), depression symptoms (29% vs. 11%, *p* = 0.003), and significant pain (38% vs. 22%, *p* = 0.01).

**Table 4:** Change in impairment of patient-reported measures in surviving multiple myeloma (MM) patients and HRS participants without multiple myeloma

Characteristic	Before MM diagnosis	MM patients after diagnosis	HRS participants without MM diagnosis^a^	*p* value
Physical impairment				
Difficulty with any ADL	15 (23%)	26 (40%)	87 (27%)	0.04
Difficulty with any IADL	19 (30%)	26 (41%)	95 (32%)	0.21
Difficulty with walking several blocks	19 (30%)	38 (60%)	119 (37%)	< 0.001
Difficulty with climbing one flights of stairs	15 (25%)	28 (47%)	91 (29%)	0.005
Sensory impairment				
Vision impairment	20 (30%)	20 (30%)	87 (26%)	0.52
Hearing impairment	13 (20%)	22 (33%)	94 (29%)	0.44
Patient experience				
Significant pain	17 (27%)	24 (38%)	69 (22%)	0.01
Poor self-rated health	24 (38%)	39 (61%)	128 (39%)	0.001
Depression	6 (13%)	14 (29%)	17 (11%)	0.003

^a^Each multiple myeloma patient was matched to 5 HRS participants without multiple myeloma diagnosis based on age, gender, race/ethnicity, marital status, education, wealth, number of comorbidities, and smoking status (using propensity score for multiple myeloma diagnosis) and exact matching on year of interview before and after diagnosis, and the presence of each patient-reported measures of interest at the interview before the multiple myeloma diagnosis of the patient with multiple myeloma

## Discussion

Using Medicare claims data linked to 26,044 HRS subjects, we identified 105 patients with multiple myeloma, 92 of which completed HRS interviews within 3 years of diagnosis. Our study showed that a multiple myeloma diagnosis negatively affects several aspects of patients’ well-being. Decline in several, but not all, patient-reported measures was significantly larger among surviving multiple myeloma patients than the changes among similar participants without multiple myeloma diagnosis. These findings have a number of limitations imposed by the nature of the HRS data, yet also provide new insights beyond what is possible with primary data collection.

Health and Retirement Study was not collected for the purpose of learning about multiple myeloma, yet the design and richness of HRS study makes it possible to describe important aspects of the patient experience. While the multiple myeloma sample in our study is not a large sample that allows for multivariate modeling, it is large enough for exploratory analyses and meaningful description of changes in impairments that matter to patients before and after diagnosis of multiple myeloma. We obtained this sample using significantly less resources and time than would be required to recruit the same number of multiple myeloma patients. For example, a primary data collection study that lasted from 2012 to 2015 recruited only 40 older patients [5], while another study recruited 41 adult patients of all ages in 3 months [4]. Furthermore, by leveraging the longitudinal nature of the HRS, our study was able to obtain data on patient-reported measures of interest prior to diagnosis, which would be impossible to obtain using primary data collection. Our study shows that we can use alternative data sources, such as claims data or larger observational studies, to design and perform some basic, but important, health research studies on rare diseases.

Our study indicates significant decline in several patient-reported measures in multiple myeloma patients after diagnosis. First, our study shows that surviving patients experience significant functional decline after the diagnosis, as measured by difficulty with activities of daily living and mobility. Prior studies in older patients with other types of cancer have also shown the functional status declines after cancer diagnoses [24, 25]. While decline in those domains is common in ageing persons, we have also shown that the functional decline in multiple myeloma patients is significantly higher than decline in similar HRS participants without multiple myeloma diagnosis. Our study also shows that there is significant decline in patient well-being, as assessed by pain, self-reported health, and depression. Similar to functional decline, reports of pain, poor or fair self-rated health, and depressive symptoms become more common in surviving multiple myeloma patients after diagnosis than participants without multiple myeloma. These results are analogous to results of another study based on secondary longitudinal data source (English Longitudinal Study of Ageing), which showed that health and well-being of patient with cancer of any type deteriorated more rapidly than health and well-being of similar patient without cancer diagnosis [26]. Our findings are significant, since impairments in well-being can themselves lead to further bad outcomes in older patients [4, 27, 28].

The measures of function and well-being that we considered in this study are rarely targets of therapy in older persons with cancer and seldom used as outcome measures in clinical trials of treatments for myeloma and other cancers. Yet these measures are very important to cancer patients, some of whom view quality of life as a more important outcome than survival. The use of population-based data sources such as the HRS to determine which measures of function and well-being are mostly likely to deteriorate after a cancer diagnosis may be useful in helping designers of cancer treatment trials consider the types of outcome measures that might be included in their trials. Furthermore, both the high mortality rate of multiple myeloma patients (20% 1-year mortality) and the high likelihood of worsening quality of life in patients with multiple myeloma highlights the importance of holistic assessment and appropriate geriatric and palliative care. Palliative care has been recognized as an important part of care for cancer patients [29, 30], where patients’ physical, mental, and psychosocial needs need to be addressed in addition to treating the underlying cancer. Unfortunately, a prior study has shown that patients with hematological cancers are less likely to receive palliative care than patients with other cancers [31].

It is important to discuss several issues in this study that can limit the clinical conclusions of this and other studies using this type of secondary data. First, the time between multiple myeloma diagnosis and the interview before the diagnosis varies from 8 days to 34 months. Since patient-reported measures are not always measured immediately before the diagnosis, patient-reported measures might have changed for some patients between the last measurement and multiple myeloma diagnosis. Similarly, the time between the diagnosis and the interview after the diagnosis also varies. The patient-reported measures are likely to change often as the treatments progress, including repeated periods of improvement and decline [6]. Second, we do not have information on the treatments that each patient is receiving, which prevents us from understanding how different treatments affect the outcomes, and when in the treatment cycle the changes occur. Thus, our results further highlight the importance of including this important information about patient oriented outcomes in observational and clinical studies of cancer therapeutics.

Our study showed that secondary data can be used to identify patients with rare diseases, and perform exploratory and hypotheses generating studies. We showed that patient-reported measures in older adults worsen after multiple myeloma diagnosis, and the decline in surviving multiple myeloma patients is significantly worse than the decline is similar older adults without multiple myeloma. This indicates that older patients could benefit from supportive geriatric and palliative care to help manage the symptoms that worsen with multiple myeloma diagnosis and treatment.
